# How will genomic information become integrated into the health care system?

**DOI:** 10.1002/mgg3.26

**Published:** 2013-07-08

**Authors:** Uta Francke

**Affiliations:** Departments of Genetics and Pediatrics, Stanford University School of MedicineStanford, California

**Figure 1:**
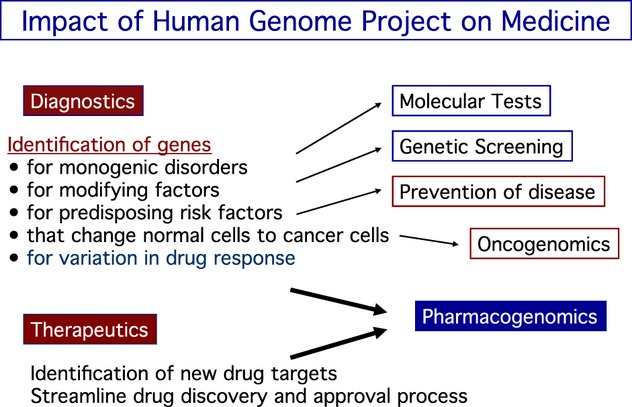
Expected impact of the human genome project on medicine, as envisioned in 2003.

On the occasion of the 60th anniversary of the publication of the DNA double helix and the 10th anniversary of the completion of the Human Genome Project, the value of genomic information to medicine continues to be widely debated. When the human genome was first sequenced it provided a “reference sequence,” an incomplete composite based on DNA from many different individuals, and the promise that it would revolutionize medical research and clinical practice. Figure 1 represents a slide that I made in 2003 summarizing our expectations at that time. Only in the last few years has the development of microarray and rapid massive parallel sequencing technologies led to breakthroughs in several areas.

Mutation detection for clinical diagnostic purposes is rapidly evolving from single disease gene testing to candidate-gene panels, to whole-exome sequencing (WES), and on to whole-genome sequencing (WGS). I have no doubt that highly automated WGS will supersede WES because it offers a more complete coverage of the genome, including all exons – beyond those captured on current exon arrays – all regulatory regions and noncoding RNA genes. Academic and commercial company scientists are making rapid progress in the filtering, interpretation, and annotation of WGS data.

As newborn screening has expanded from a handful of tests to quantitative data on compounds of known or unknown identity by tandem mass spectrometry, a global method, so will WGS be done on cord blood samples routinely collected at the time of delivery. It has been proposed that the information be stored and scrutinized over time for age-appropriate information. I would argue, however, that the discovery of highly penetrant BRCA or other cancer gene mutations via newborn screening is of great relevance for adult relatives who may be at high cancer risk without knowing it. The highly charged ethical debate about which “incidental findings” – discovered via a physician-ordered sequence-based diagnostic test – to share with patients and their families will become moot when in newborn WGS screening, or in screening of healthy individuals in general, all abnormal results are “incidental” and generalized standards for informed consent and appropriate counseling will be in place.

Looking ahead, as genome sequencing becomes widely available, how will genomic information affect the theory and practice of medicine? First, I do not think we need a new genetics subspecialty called “genomic medicine” or a “clinical genomicist” as a new professional specialist. Another model would be an interdisciplinary clinic staffed by experts in medical genetics, genetic counseling, molecular genetics, bioinformatics, and bioethics, possibly with individual members able to cover more than one area. While academic medical centers may experiment with establishing such “Genomic Medicine” clinics where people can take their genome data and receive interpretation, counseling, and referrals to disease-specific medical specialists, this approach does not seem scalable, mostly for manpower and reimbursement reasons.

In reality, I believe genomics will enter medicine, as other new technologies have done, one specialty at a time, with variable speed and impact. Eventually, all healthcare providers will have to learn to incorporate genomic data, together with clinical, imaging, and standard laboratory data, in work-up and treatment plans. The newly discovered abundance of genome variants, copy number variants (CNVs) and single nucleotide variants (SNVs) – with detrimental, benign, or unknown effects on gene function and phenotype – are best interpreted in the context of medical knowledge of the disease and the individual's personal and family medical history. Risk prediction algorithms incorporating polygenic scores, as well as diagnostic and therapeutic decision trees, will be developed by panels of experts to help the front-line clinicians. Genomic data provide genetic risk estimates. But most diseases result from the combination of genetic and environmental factors, in an additive or interactive fashion. Therefore, knowing one's genetic risk is a great motivator for life-style changes with the goal of minimizing nongenetic risk factors.

Oncology is the leading specialty in applying new cancer genomics information. The number of genes and pathways implicated in the predisposition and development of neoplasias is truly staggering. Tumorigenesis can be driven by mutations in one or more of ∼140 different genes that function in 12 different signaling pathways controlling cell growth, survival, and genome maintenance (Vogelstein et al. 2013). Inherited and somatic mutations found in individual cancers are not completely random but are cell-type rather than organ-type specific. Cancers will be classified and subclassified by their genomic profiles rather than by the anatomic site of the tumor. Such classification can be done even at the level of a single cell (Zong et al. 2012). The genomic and transcriptomic profiles will contribute to the determination of prognosis and treatment choices.

The cancer genomics discoveries lead to the promise of “precision” therapy that is specifically targeted to the individual patient's cancer. An initial success story is the development of BRAF inhibitors for the treatment of malignant melanoma. Fifty percent of cutaneous melanomas have specific mutations in BRAF (mostly V600E and less often V600K) that increase the activity of the mitogen-activated protein kinase signaling pathway. Following treatment with drugs that specifically inhibit mutant BRAF, tumor and metastases literally melted away. But the disease invariably relapsed a few months later (Flaherty et al. 2010; Sosman et al. 2012). As cancer genomes are unstable and prone to mutagenesis, malignant subclones arise that are resistant to the previous therapy. The recent discovery of tumor DNA in circulating blood promises that relapses and newly arisen mutations will be detected and monitored by sequencing plasma-derived DNA (Forshew et al. 2012). By using this noninvasive method to assess the level and type of mutations in circulating tumor DNA, treatment protocols could be adapted to the individual patient's genomic profile.

The opportunity to develop drugs that target specific mutations in specific tumor-driving genes is intellectually exciting, but there are concerns about feasibility and cost, given the expense of bringing new drugs to market. Gleevec, a drug that is targeting the product of a specific chromosomal rearrangement, is the poster child of this approach. The cost of treatment, however, exceeds by far the utility, with millions of affected people around the world unable to afford the excessive price (Pollack 2013). Given the experience with BRAF inhibitors and Gleevec, the future of most cancer treatment in adults could be envisioned as a chase to detect ever-changing somatic mutations and apply combination therapies to gain a few months of life for affected individuals, at a considerable cost to the health care system. An alternative or complementary treatment strategy that is also actively pursued at this time aims to strengthen the patient's own immune system to fight cancer of any type.

More gain in reducing the morbidity and mortality from cancer could be made in the area of prevention, in particular, where environmental exposure factors are clearly established, such as for smoking and lung cancer, and where other preventive steps, such as risk-reducing surgery, can be taken. Genomics will contribute to the identification of heritable germ-line mutations that predispose to cancer, for example, colorectal, breast, ovarian, and pancreatic cancer. Currently, testing for highly penetrant inherited cancer mutations is recommended only for family members of affected individuals. In the future, when genotyping and genomic sequencing data will be widely available, a population-based prevention approach could be developed. Millions of people who are genetically predisposed to a cancer that they do not yet have will benefit by taking advantage of preventive measures, as will their close relatives who are alerted to the fact that a cancer gene is in their family. Our small study of the reactions of BRCA1 and BRCA2 mutation carriers, identified through a direct-to-consumer genotyping service, supports this prediction (Francke et al. 2013).

Genomic technology is driving a major advance in prenatal medicine. The surprising discovery of abundant short fragments of fetal DNA in maternal blood throughout pregnancy has opened up new avenues for noninvasive prenatal testing. Only a few years after the initial research discovery (Fan et al. 2008), noninvasive prenatal testing/screening (NIPT) for aneuploidy by sequencing cell-free fetal DNA (cff DNA) in maternal blood has been approved for use in the clinic (ACOG Committee on Genetics 2012). This initiated a fierce competition between four test-providing companies (Weaver 2013). With a high level of accuracy and very low rate of false-negative results, the noninvasive cff DNA in maternal blood test will replace chorionic villus sampling and amniocentesis as screening tests for high-risk pregnancies. While at present a positive result requires confirmation by amniotic cell studies, the false-positive rate is likely to drop to essentially zero with increasing experience, and then confirmatory testing would no longer be required. I do remember that in the early days of prenatal diagnosis by amniocentesis, fetal blood sampling from placental vessels was carried out to confirm a positive finding. In the future, I envision that NIPT costs will drop and maternal blood screening for fetal chromosome imbalances will become available for pregnant women of all ages.

Noninvasive first trimester fetal testing for inherited disorders will become possible as well, initially for autosomal dominant disorders when the father carries a known disease-causing mutation. For X-linked recessive disorders, maternal blood screening for fetal sex could identify female fetuses who would not have to undergo further invasive testing. Once mutation-bearing DNA fragments can be accurately quantified in cff DNA, fetuses carrying a maternally inherited mutation could also be identified. This will increase the scope of NIPT to include prenatal diagnosis of affected boys with an X-linked disorder and of all offspring who inherited recessive alleles from both parents. In all these high-risk pregnancies, the shortened turn-around time and less invasive procedure will be a great advantage.

**Figure 2:**
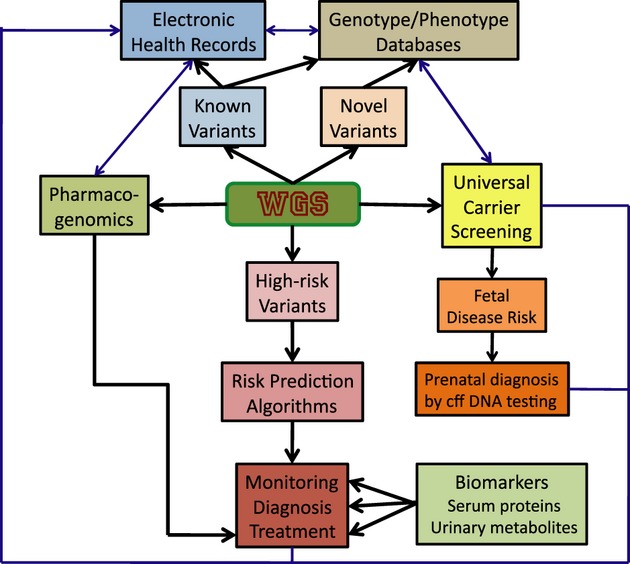
Model for integration of WGS data into diagnostics and therapeutics, genotype and phenotype databases and electronic health records. Two-sided arrows indicate reciprocal communication between sets of data.

A comprehensive vision of the integration of WGS data into an interactive scheme is shown in Figure 2. The model is DNA-centric for reasons of simplicity, and is not meant to exclude the impact of transcriptomic, metabolomic, and proteinomic information that will become increasingly available as well.

Starting in 2007, genome-wide association studies led to a spectacular rise in knowledge regarding associations between SNVs and common disease phenotypes, thereby revolutionizing research on these disorders. While small effect sizes of individual SNV genotypes limit their usefulness for clinical predictions, associations between diseases and SNVs in or near genes that were not previously known to be linked with those diseases are being discovered. Such discoveries provide new insights into genes and pathways important for pathogenesis and may yield novel drug targets.

Furthermore, certain SNVs are found to be associated with multiple diseases, for example, various autoimmune disorders, pointing to common underlying mechanisms. In a recent meta-analysis of five different psychiatric disorders (autism spectrum, attention deficit hyperactivity disorder, bipolar and major depressive disorder, and schizophrenia) the Cross-Disorder Group of the Psychiatric Genomics Consortium (2013) discovered significant associations of SNVs with more than one of these disorders. Some of these cross-disorder SNVs are in or near genes that affect calcium channel activity. These data call into question the validity of strict diagnostic categories and suggest etiologic overlap. They provide support for a paradigm shift from the age-old classification of psychiatric disorders into a new nosology that uses genomic and causative information. Physicians and patients already know that symptoms can overlap and extend across diagnostic boundaries.

Another emerging theme is that “common diseases” may not be single disorders at all, but include a collection of rare disorders with similar overlapping phenotypes that are distinguished by the underlying genetic causation. As genomic information infiltrates various areas of medicine, it will lead to fundamental changes in disease classification, diagnostic concepts, and therapeutic approaches.

Finally, the patient–doctor relationship will be drastically changed by the consumer empowering movement. As people gain greater access to their health information, including genomic information, and to tools allowing them to monitor their health parameters, they will increasingly become partners in managing their health. We are on a fast-moving train and the destination may surprise us.
